# Monitoring of Peroxide in Gamma Irradiated EVA Multilayer Film Using Methionine Probe

**DOI:** 10.3390/polym12123024

**Published:** 2020-12-17

**Authors:** Nina Girard-Perier, Magalie Claeys-Bruno, Sylvain R. A. Marque, Nathalie Dupuy, Fanny Gaston, Samuel Dorey

**Affiliations:** 1 Sartorius Stedim FMT S.A.S, Z.I. Les Paluds, Avenue de Jouques CS91051, 13781 Aubagne CEDEX, France; nina.girard@sartorius.com (N.G.-P.); fanny.gaston@Sartorius.com (F.G.); 2 Aix Marseille Univ, Avignon Université, CNRS, IRD, IMBE, 13013 Marseille, France; 3 Aix Marseille Univ, CNRS, ICR, Case 551, 13397 Marseille, France

**Keywords:** methionine oxidation, radicals, liquid chromatography, chemometrics, single-use bag, design of experiments

## Abstract

In this study, the oxidation of methionine is used as a proxy to model the gamma radiation-induced changes in single-use bags; these changes lead to the formation of acids, radicals, and hydroperoxides. The mechanisms of formation of these reactive species and of methionine oxidation are discussed. With the help of reaction kinetics, the optimal conditions for the use of these single-use bags minimizing the impact of radical chemistry are highlighted. Biopharmaceutical bags gamma irradiated from 0 kGy to 260 kGy and aged from 0 to 36 months were filled with a methionine solution to follow the oxidation of the methionine. The methionine sulfoxide was measured with HPLC after different storage times (0, 3, 10, 14, 17, and 21 days). Three main results were analyzed through a design of experiments: the oxidative induction time, the methionine sulfoxide formation rate, and the maximum methionine sulfoxide concentration detected. A key aspect of the study is that it highlights that methionine is oxidized not necessarily directly by hydro(gen) peroxide but throughperacid, and likely peracetic acid. The answers to the design of experiments were considered to obtain the desirability domain for the optimization of the conditions of use for the single-use bags limiting the oxidation of methionine as well as the release of reactive species thereof.

## 1. Introduction

The use of single-use bags brings significant improvement in the manufacturing of biopharmaceutical molecules, as the cleaning validation [1] step is suppressed, leading to savings of time and money. Moreover, single-use significantly reduces the risks of contamination. Compared with traditional stainless steel containers, single-use bags also increase handling facility and productivity [2,3]. These technologies become more important as biopharmaceutical manufacturers are facing increasing pressure to reduce product costs while maintaining a high-quality product. Single-use bags are mainly manufactured from appropriate multilayer polymer films with a gas barrier layer such as ethyl vinyl acetate/ethyl vinyl alcohol/ethyl vinyl acetate (EVA/EVOH/EVA) or polyethylene/ethyl vinyl alcohol/polyethylene (PE/EVOH/PE). Bags should be sterilized before being used and gamma irradiation is the conventional method of radiation sterilization. The gamma irradiation dose range usually used for biopharmaceutical industries is between 25 and 45 kGy [4]. The sterilization process is essential, but not without consequences. Indeed, many studies report on the modification of these single use systems after sterilization. One of these degradations, which represents a key concern when leaching out in biopharmaceutical solutions, and can generate extractables or leachables [5]. Moreover, the process of sterilization can induce modifications in the materials [6]. Gamma sterilization of single-use systems initiates chemical reactions generating radicals [7] and modifications inside the material affording changes in the molecular weight of polymers [8]. The gamma irradiation-induced chemical modifications of an EVA/EVOH/EVA multilayer film have been thoroughly studied using various techniques (pH analysis [9], FTIR spectroscopy [10], X-ray photoelectron spectroscopy [11] for the in-depth and surface detection of oxidized species in materials. The generation of radicals, and thus the polymer modifications, can induce protein aggregation and protein oxidation [12,13]. This study is focused on the oxidation of the methionine stored in single-use systems made from an EVA/EVOH/EVA multilayer film. Methionine is very sensitive to radicals, particularly to reactive oxygen species (ROS), and is most of the time oxidized to methionine sulfoxide. This oxidized form can inactivate proteins and cause a decrease in biological activity [14]. We chose to study methionine oxidation as a model for protein oxidation as methionine is one of the main amino acids prone to oxidation; this high reactivity is due to the reaction of the sulfur atom with oxygen species [15,16].

Although methionine oxidation is comprehensively studied in the literature, there is no publication on the oxidation of methionine in biopharmaceutical bags. Only one paper related to medical devices has been found. Masato et al. [17] showed that the oxidation of methionine under thermal or light stress disappears when a therapeutic antibody is filled into a polymer-based syringe along with an oxygen absorber.

The aim of our work was, therefore, to establish the conditions for the oxidation of methionine according to bag aging and irradiation doses to highlight the presence of reactive species. The methionine sulfoxide concentration was quantified using high-performance liquid chromatography (HPLC), and all data were analyzed through a design of experiments [18,19,20,21,22]. The final objective was then to optimize the single-use biopharmaceutical bags usage to limit the oxidation of methionine and the release of reactive species thereof.

## 2. Materials and Methods

### 2.1. Sample Bags

**Figure 1:**
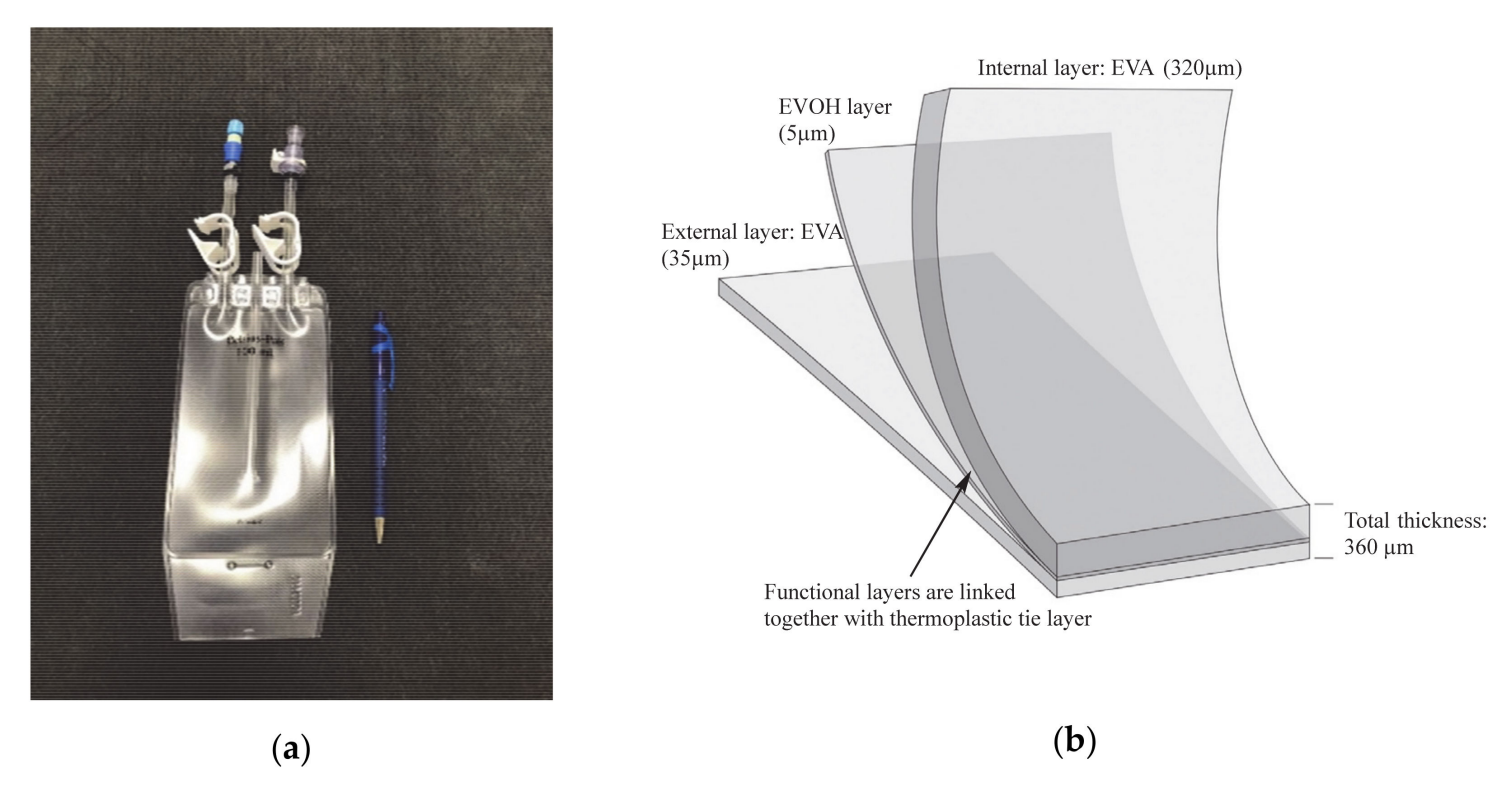
EVA single-use plastic bag (**a**) and composition of the EVA multilayer film (**b**).

The two lots of EVA single-use plastic bags investigated (Figure 1a) were made from a multilayer film composed of one layer of ethylene vinyl alcohol (EVOH) sandwiched between two layers of ethylene vinyl acetate (EVA), with a total thickness of about 360 µm (Figure 1b). Sample bags have been provided by Sartorius Stedim FMT S.AS, Aubagne, France.

### 2.2. Gamma Irradiation

EVA plastic bags were packed, wrapped in multilayer packaging (polyethylene/polyamide/polyethylene) and irradiated at room temperature with a ^60^Co gamma source at Ionisos, Dagneux, France. The target dose averages reached were 29 kGy, 59 kGy, 106 kGy, and 260 kGy. The dose rate provided was of 1–2 kGy/h. Irradiation was performed at room temperature under an environmental atmosphere.

### 2.3. Ageing and Storage Times

**Figure 2:**
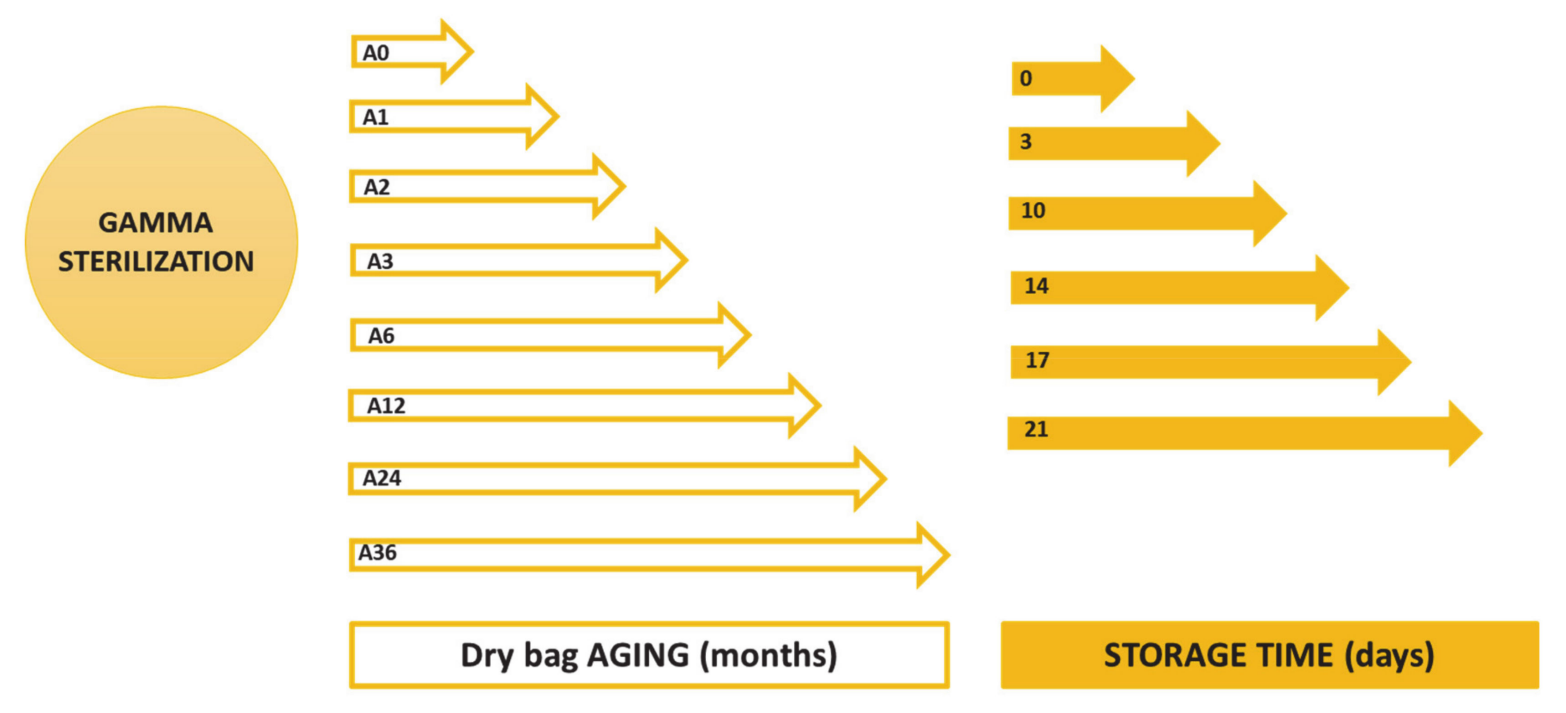
Aging and storage time details.

The sample bags were analyzed at different times after gamma irradiation. Two times were considered (Figure 2): “aging” for the time between irradiation and filling of the bag and “storage” for the time of storage of the methionine solution in the bag after the end of each aging time. Eight aging times (A) were considered: A0, A1, A2, A3, A6 months at room temperature and 130 days, 235 days, and 365 days at 40 °C to simulate A12, A24, and A36 months, respectively. These corresponding times were calculated based upon the ASTM F1980 regulation [23] using Q10 = 2. Six storage times were considered: 0, 3, 10, 14, 17, and 21 days. Finally, a full factorial experimental design was built (see Section 2.8 for details).

### 2.4. Chemicals and Reagents

Methionine (purity, 98.0%), methionine sulfoxide (purity, 99.0%), sodium phosphate monobasic (purity, 98.0%), sodium tetraborate decahydrate (purity, 99.5%), sodium azide (purity, 99.5%) and L-Norvaline (purity, 99.0%) were purchased from Sigma Aldrich, St Quentin Fallavier, France.

HPLC-grade acetonitrile and methanol were from Sigma Aldrich. Water for HPLC was purified using the Milli-Q purification system by Merck Millipore, Darmstadt, Germany. The OrthoPhtalAldéhyde (OPA) and borate reagent used for derivatization were purchased from Agilent, Waghaeusel-Wiesental, Germany.

### 2.5. Methionine Solution Filling

Each bag (for each aging, each lot, and each dose) were filled with a 50 µM solution of methionine in buffer (10 mM NaH_2_PO_4_, 10 mM Na_2_B_4_O_7_•10H_2_O, 5 mM NaN_3_, pH 8.2).

### 2.6. HPLC System and Conditions

To determine the concentration of methionine and methionine sulfoxide, an Agilent 1260 HPLC (Waghaeusel-Wiesental, Germany), equipped with a quaternary pump (G1311C), an autosampler (G1329B), and a fluorescence detector (G1321B), was used. Before analysis, the flow rate was set to 3.0 mL/min using vacuum-degassed mobile phases (A, 10 mM NaH_2_PO_4_, 10 mM Na_2_B_4_O_7_•10H_2_O buffer at pH = 8.2; B, acetonitrile:methanol:water (45:45:10, *v:v:v*)). Before use, solvent A was filtered through a 0.22 μm microporous cellulose acetate filtering membrane. The automated online derivatization (in the autosampler) using an injection program is detailed in Table S1. The derivatization reagent used was the ortho-phthaldehyde (OPA).

The gradient program was as follows: 0–13.4 min, 2% phase B; 13.4 min, 57% phase B; 13.5–15.8 min, 100% phase B; 18 min, 2% phase B. Separation was carried out on an Agilent Poroshell HPH-C18 column (4.6 mm × 100 mm, 2.7 μm particles—Waghaeusel-Wiesental, Germany) used with a pre-column, UHPLC guard Poroshell HPH-C18, 4.6 mm. The column was maintained at 40 °C ± 0.8 °C in a thermostatted column compartment (G1316A) during the analyses. The fluorescence detector was set to an excitation wavelength of 340 nm and an emission wavelength of 450 nm. The total runtime of the method was 20 min. Chromatographic data were acquired and evaluated with the HPLC 1260 OpenLAB software (Waghaeusel-Wiesental, Germany). Internal calibration was done by spiking 20 µL of L-Norvaline in each sample and standard.

### 2.7. Results Treatment

The limit of detection (LOD) and limit of quantification (LOQ) were determined after five repeated runs of low concentration levels (0.25–1.1 µmol/L) of standard solutions, which generated a signal to noise ratio of 3 for LOD and a signal to noise ratio of 10 for LOQ. The LOD was 0.15 μM; while the LOQ was 0.25 μM.

### 2.8. Design of Experiments

In order to compare all conditions (in terms of aging and dose) of methionine oxidation into methionine sulfoxide, a design of experiments (full factorial) was performed with the Azurad software [24] with a [4 × 8] matrix.

#### 2.8.1. Factors and Experimental Domain of Interest

The gamma irradiation dose (X1) and the aging time (X2) are two essential parameters in the study of methionine oxidation. The experimental domain for each parameter is described in Table 1.

#### 2.8.2. General Protocol

**Figure 3:**
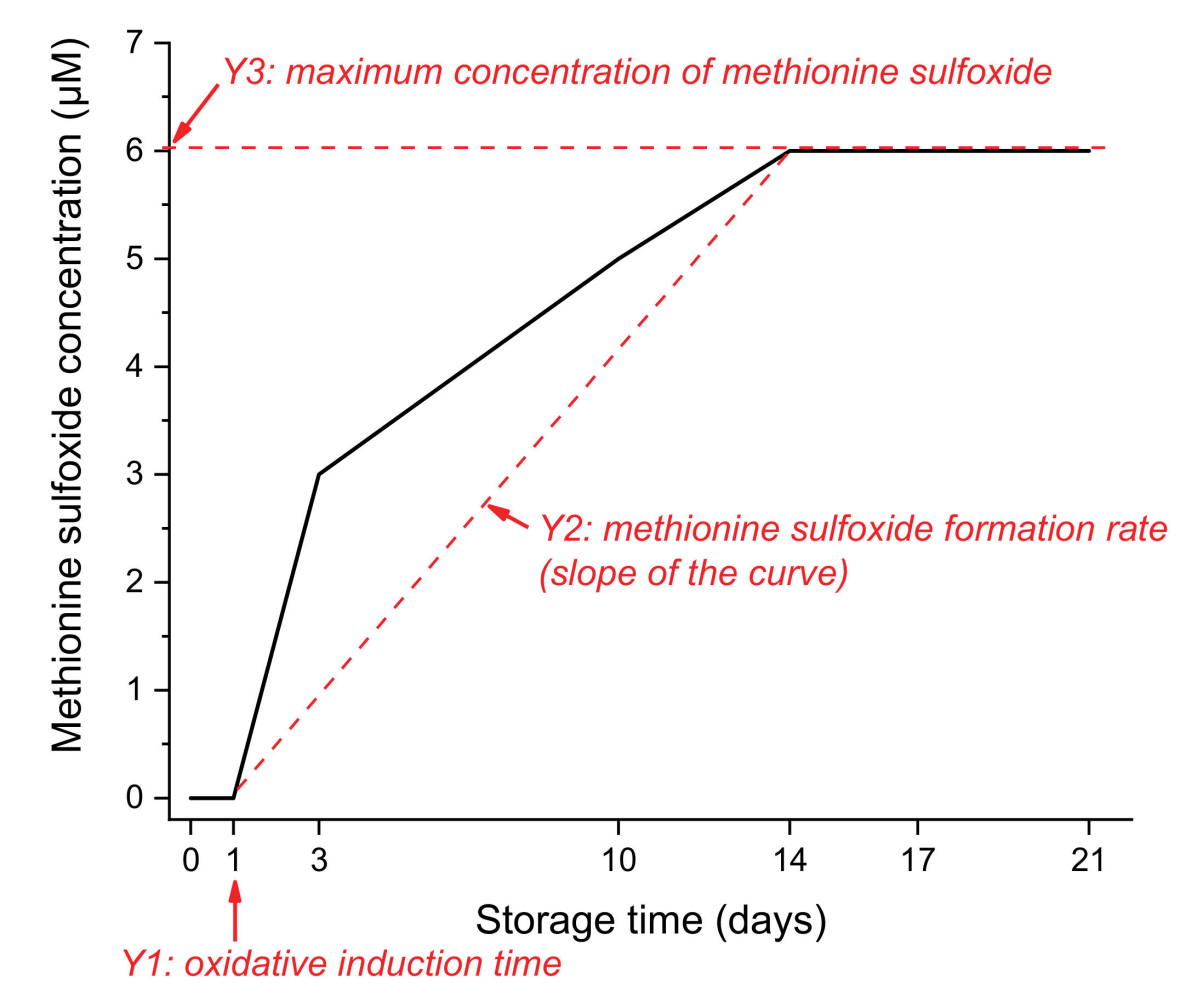
A plot of storage time vs methionine sulfoxide concentration generated to exemplify the three responses used in the experimental design.

Once the sample bag has been gamma-irradiated, the dry aging starts (from 0 to 36 months). After each of these dry aging periods, the bag is filled with a methionine solution and methionine sulfoxide is measured by HPLC after different storage times (0, 3, 10, 14, 17, and 21 days). We can thus obtain the curve, shown in Figure 3, representing the methionine sulfoxide concentration as a function of the storage time. In the aim to simplify the processing of the results and find the conditions to minimize the methionine oxidation, three responses were studied:

- Y_1_: the oxidative induction time in days. For example, in Figure 3, the oxidative induction time for the red curve is 1 day.
- Y_2_: the methionine sulfoxide formation rate represented by the slope of the methionine sulfoxide concentration-storage time curve. For example, in Figure 3, the slope of the red curve is obtained with x and y values at 1, 3, and 10 days.
- Y_3_: the maximum concentration of methionine sulfoxide detected in µM. This response is obtained by averaging the four concentrations obtained at 10, 14, 17, and 21 days.

## 3. Results

### 3.1. Methionine Oxidation Conditions

The methionine can be oxidized in methionine sulfoxide (mono-oxidation) and then in methionine sulfone (double oxidation) [25,26]. The conditions of methionine oxidation in the presence of hydrogen peroxide and acetic acid were firstly checked (Table 2) and monitored over time. Only methionine sulfoxide was detected in our experimental set.

The methionine sulfoxide concentration was measured by HPLC at 0, 3, 14, and 21 days. Results are available in Table 2. As expected, methionine is not oxidized by acetic acid (test 3). For all other tests, the methionine oxidation increases with time. After 21 days, the highest methionine sulfoxide concentration is observed for test 7, when the non-sterile bag is filled with hydrogen peroxide and methionine in stoichiometric conditions (50 µM), reaching 26 µM.

The second-highest concentration is observed for test 4, when the beaker is filled with 30 µM hydrogen peroxide and 100 µM methionine, reaching 23 µM. These two tests highlighted that hydrogen peroxide oxidized the methionine, as expected. The slightly larger oxidation of methionine in the bag (test 7) than in the beaker (test 1) was likely due to the process of degradation of the bag generating carboxylic acid and H_2_O_2_. Indeed, as is well known in the literature, a solution of H_2_O_2_ and acetic acid to generate in situ peracetic acid efficiently oxidizes a solution of methionine, as highlighted in Figure S3 (tests 2 and 5). To further confirm that peracetic acid is more efficient at oxidizing methionine than hydrogen peroxide, we carried out the experiment proposed by Du et al. [27] adjusting concentrations so that they were in excess. We added acid acetic at 256 mM, hydrogen peroxide at 76 mM with 50 µM of methionine. From the results in Table 2, we clearly saw that methionine was immediately (within an hour) oxidized in methionine sulfoxide.

### 3.2. Methionine Oxidation Kinetics

The results of the design of experiments are detailed in Table S2.

#### 3.2.1. Evolution of the Oxidative Induction Time (Y1)

**Figure 4:**
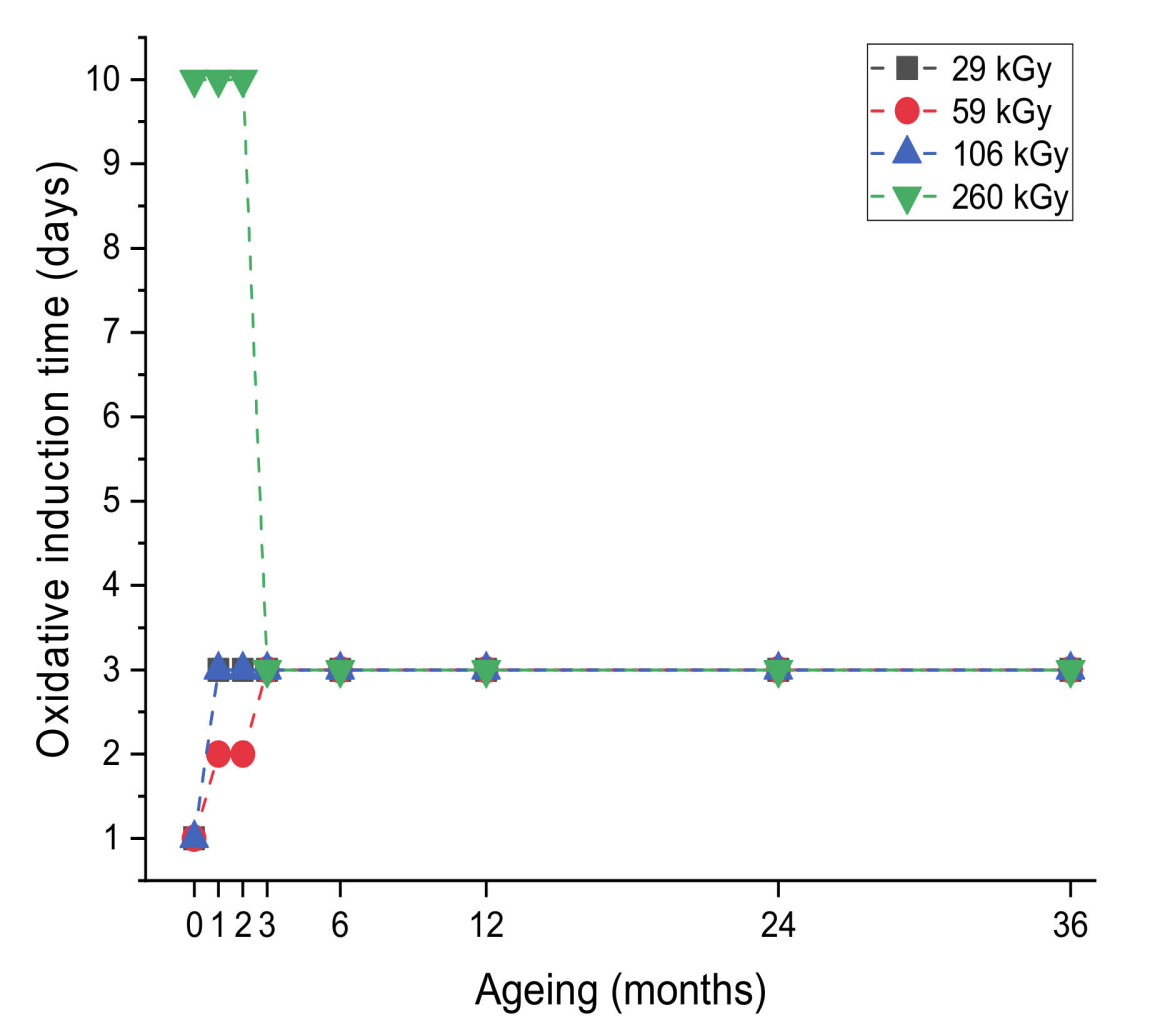
Plots of aging before filling versus induction time for the oxidation of methionine at different doses.

Figure 4 shows the oxidative induction time as a function of the aging time (time gap between irradiation and filling). There were two different oxidative induction time behaviors, caused, on the one hand, by the 29, 59, and 106 kGy doses and, on the other hand, by the 260 kGy dose. For each dose, two stages were observed. For doses at 29, 59, and 106 kGy, and when bags were filled immediately after irradiation (aging 0), the oxidative induction time was 0 (day). This means that methionine oxidation appeared as soon as the methionine solution was in contact with the bag materials. When bags were filled from 1 month to 36 months after irradiation, the oxidative induction time was 2–3 days. For the 260 kGy dose, the oxidative induction time displays an opposite behavior. When bags are filled up to 2 months after irradiation (0, 1, and 2 months), the oxidative induction time was 10 days, while when aging increases from 3 up to 36 months, the oxidative induction time is constant at 3 days. For this dose, methionine oxidation appeared quickly when bags were filled right after irradiation, whereas it took longer to appear when bags were aged after irradiation.

#### 3.2.2. Evolution of Methionine Sulfoxide Formation Rate (Y2)

**Figure 5:**
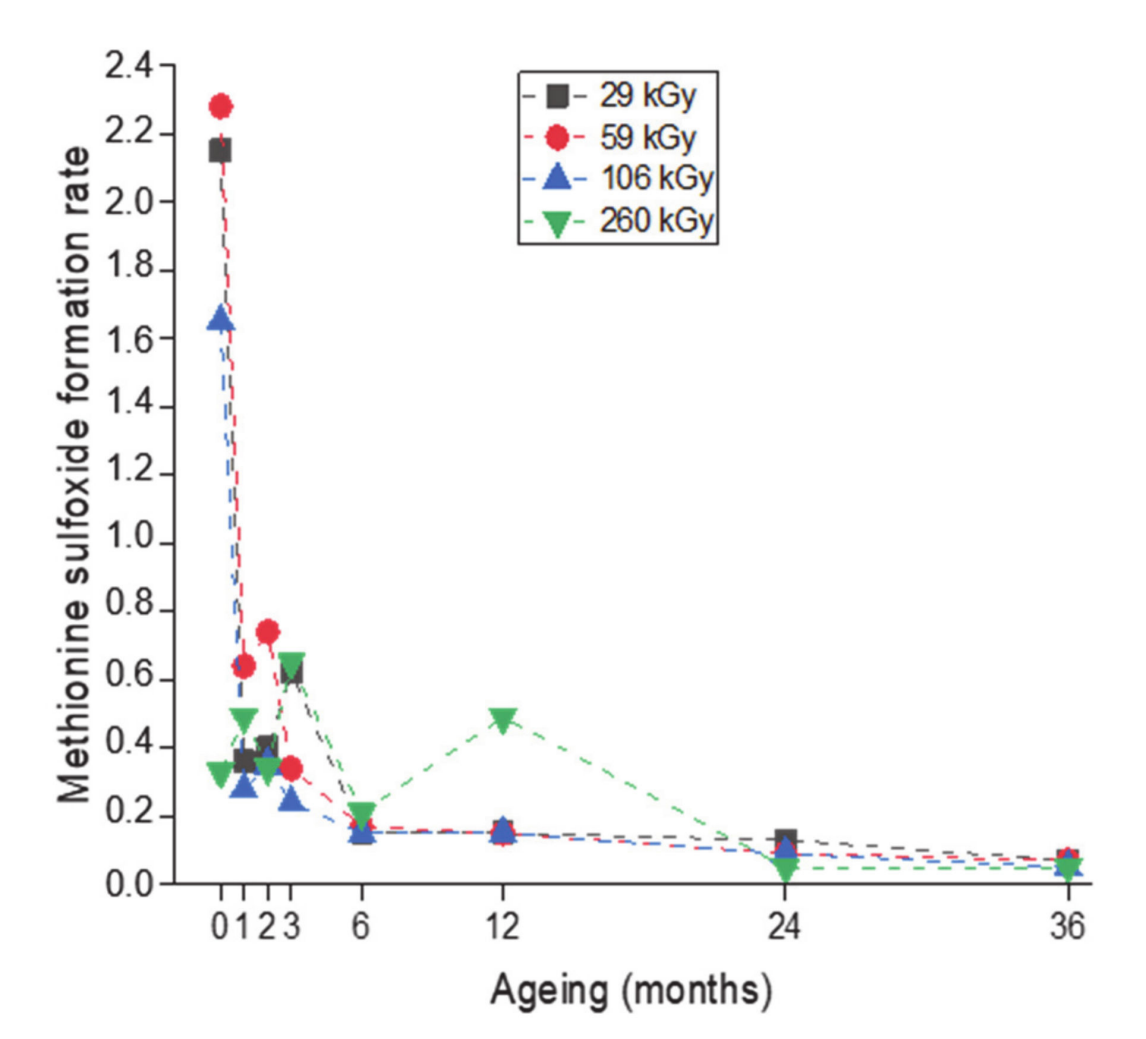
Plots of aging before filling versus methionine sulfoxide formation rate at different doses.

The behavior of the methionine sulfoxide formation rate at 29, 59, and 106 kGy is different from that at 260 kGy (Figure 5). Shortly after irradiation (aging 0), this formation rate is higher for the low doses (2.1 µM/day for 29 kGy, 2.3 µM/day for 59 kGy and 1.7 µM/day for 106 kGy bags) than for the high dose (0.3 µM/day for 260 kGy). From one month to 36 months after irradiation, we can observe a shift for the low doses (29; 59 and 106 kGy), reaching methionine sulfoxide formation rates below 0.8 µM/day.

The methionine sulfoxide formation rate for the 29, 59 and 106 kGy irradiated bags is therefore aging dependent. It is high when the aging time is short and it decreases as the aging time increases. For the 260 kGy irradiated bag, the methionine sulfoxide formation rate is independent of the aging time, as it remains low (<0.8 µM/day) whatever this time.

#### 3.2.3. Evolution of the Maximum Methionine Sulfoxide Concentration Detected (Y3)

**Figure 6:**
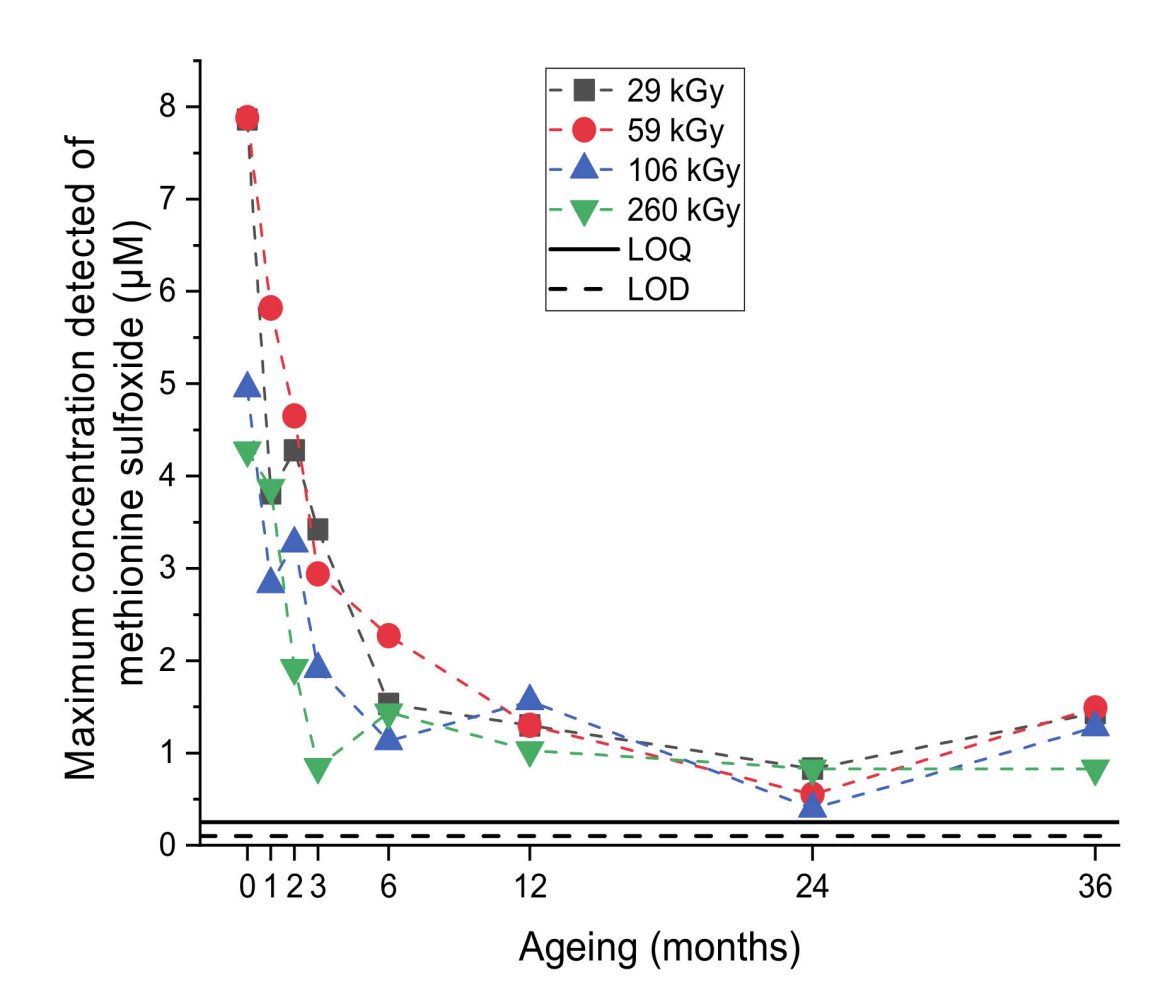
Plots of aging before filling versus maximum methionine sulfoxide concentration detected at different doses.

Quickly after irradiation (aging 0), bags irradiated at 29 and 59 kGy exhibit the highest concentration in methionine sulfoxide (~8 µM, Figure 6). Oxidation is lower for bags irradiated at 106 kGy and 260 kGy, i.e., 5 µM and 4.3 µM, respectively. For bags irradiated at doses ≤106 kGy and from one month after irradiation, the methionine sulfoxide concentration decreases to reach ~1.5 µM after 36 months of aging. For the bag irradiated at 260 kGy, from 0 to 3 months after irradiation the methionine sulfoxide concentration decreases (from 4.3 µM to 0.9 µM), then reaching a plateau at ~1 µM over time up to 36 months.

### 3.3. Modeling

#### 3.3.1. Mathematical Modeling

The three responses (Y_1_: oxidative induction time (days), Y_2_: methionine sulfoxide formation rate and Y_3_: maximum concentration of methionine sulfoxide detected (µM)) were then modeled. Based on the experiment domain displayed in Table 1, we used a polynomial model of degree 2, which can be written as:

$$Yi=b_{0}+b_{1}\times X_{1}+b_{2}\times X_{2}+b_{11}\times X_{1}^{2}+b_{22}\times X_{2}^{2}+b_{12}\times X_{1}X_{2}$$

Variables X_1_ and X_2_ are defined in Table 1, and the b_i_ and b_ij_ coefficients affording the best fit in Table 3.

From the experimental results, the model coefficients were calculated using multi-linear regression on the coded variables and are displayed in Table 3.

Most of the residues have values close to zero and are distributed on either side of the axis, which allows the model to be validated with R² equal to 0.56, 0.38, and 0.70 (with a degree of freedom = 62, 61, and 59), respectively for the three responses.

#### 3.3.2. Interpretation of Response Surfaces

##### Oxidative Induction Time (Y_1_)

**Figure 7:**
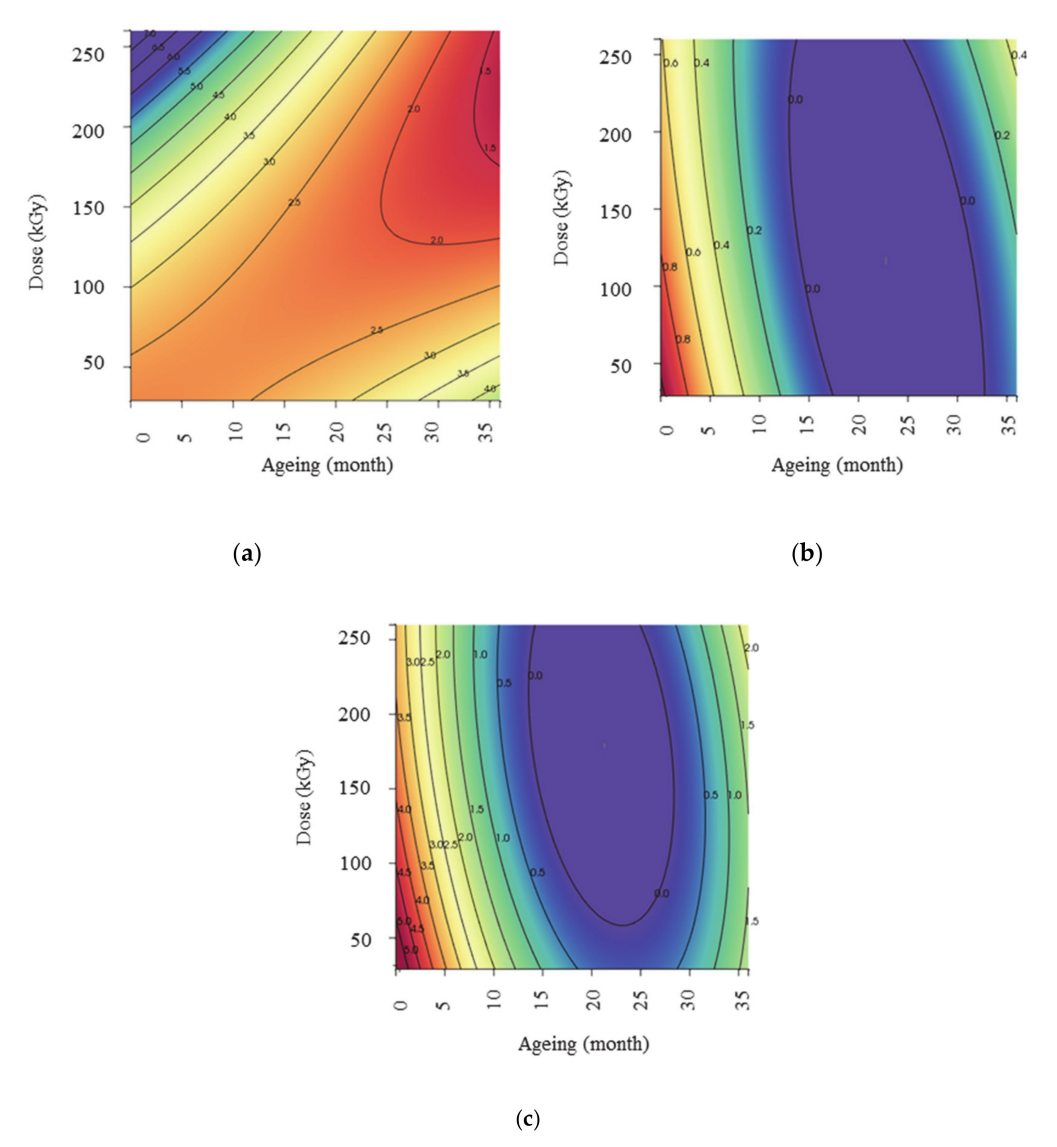
Response surface of the effect of aging (months) vs dose (kGy) (**a**) on the oxidative induction time, Y_1_ (day); (**b**) on the methionine sulfoxide formation rate Y_2_ (µM/day); (**c**) on the maximum methionine sulfoxide concentration detected, Y_3_ (µM).

Figure 7a shows the response surface of the oxidative induction time. The bluish area in the top left corner represents the conditions leading to the highest oxidative induction times, i.e., for bags irradiated at doses ~250 kGy and shortly after irradiation. For doses above 50 kGy, the earlier the bag is used after irradiation, the shorter the induction time. At the bottom right-hand corner, we can also observe high values of oxidative induction times for bags irradiated at ~50 kGy and aging >24 months. This means that for doses <100 kGy, the longer the aging (time gap between irradiation and use thereof), the longer the oxidative induction time.

##### Methionine Sulfoxide Formation Rate (Y_2_)

Figure 7b shows the response surface of the methionine sulfoxide formation rate. This formation rate is age dependent. The red area in the bottom left corner of Figure 7b represents the highest methionine sulfoxide formation rate in our model. When bags are irradiated at doses lower than 100 kGy and used shortly after irradiation (aging <5 months), the methionine sulfoxide formation is faster than for bags aged more than 10 months after irradiation.

##### Maximum Concentration of Methionine Sulfoxide Detected (Y_3_)

Figure 7c shows the response surface of the maximum concentration of methionine sulfoxide detected. The red area in the bottom left corner of Figure 7c represents the highest maximum concentration of methionine sulfoxide detected. The maximum concentration of methionine sulfoxide is detected for bags irradiated at doses lower than 100 kGy and used shortly after irradiation (aging <5 months).

## 4. Discussion

**Figure 8:**
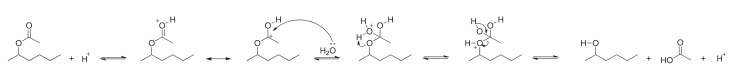
Hydrolysis of the ethylene vinyl acetate leading to carboxylic acid.

**Scheme 1:**
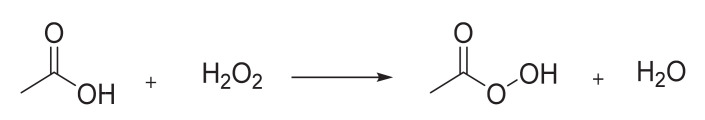
Formation of peracetic acid.

**Scheme 2:**
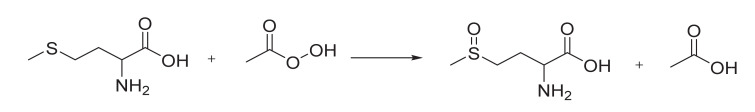
Formation of methionine sulfoxide.

The experimental results show that methionine sulfoxide is generated when a solution of methionine is stored in irradiated bags. The level of oxidation depends in our testing conditions on the irradiation dose, the bag aging before filling, and the storage time of the solution in the bag. The formation of methionine sulfoxide is obviously due to the presence of oxidants generated from the degradation of the multilayer film upon gamma irradiation. As no radical species are detected in the samples, oxidation is likely due to the presence of hydrogen peroxide, peracids, or in situ generated peracids. Model experiments (Figure S1) show that over a long time, H_2_O_2_ affords oxidation product and that in situ generated peracetic acid is also efficient at oxidizing, as expected [17,27]. Indeed, the modification of EVA/EVOH/EVA multilayer film under gamma-irradiation shows the generation of acids (Figure 8), degradation of the oxygen barrier property of the EVOH layer with increasing irradiation dose, [28] and the absence of EPR signal in the EVOH layer, meaning that the generation of a large amount of H_2_O_2_ is very unlikely. Thus, the in situ generation of peracetic acid (Scheme 1) is not the major process leading to the oxidation of methionine (Scheme 2).

**Scheme 3:**
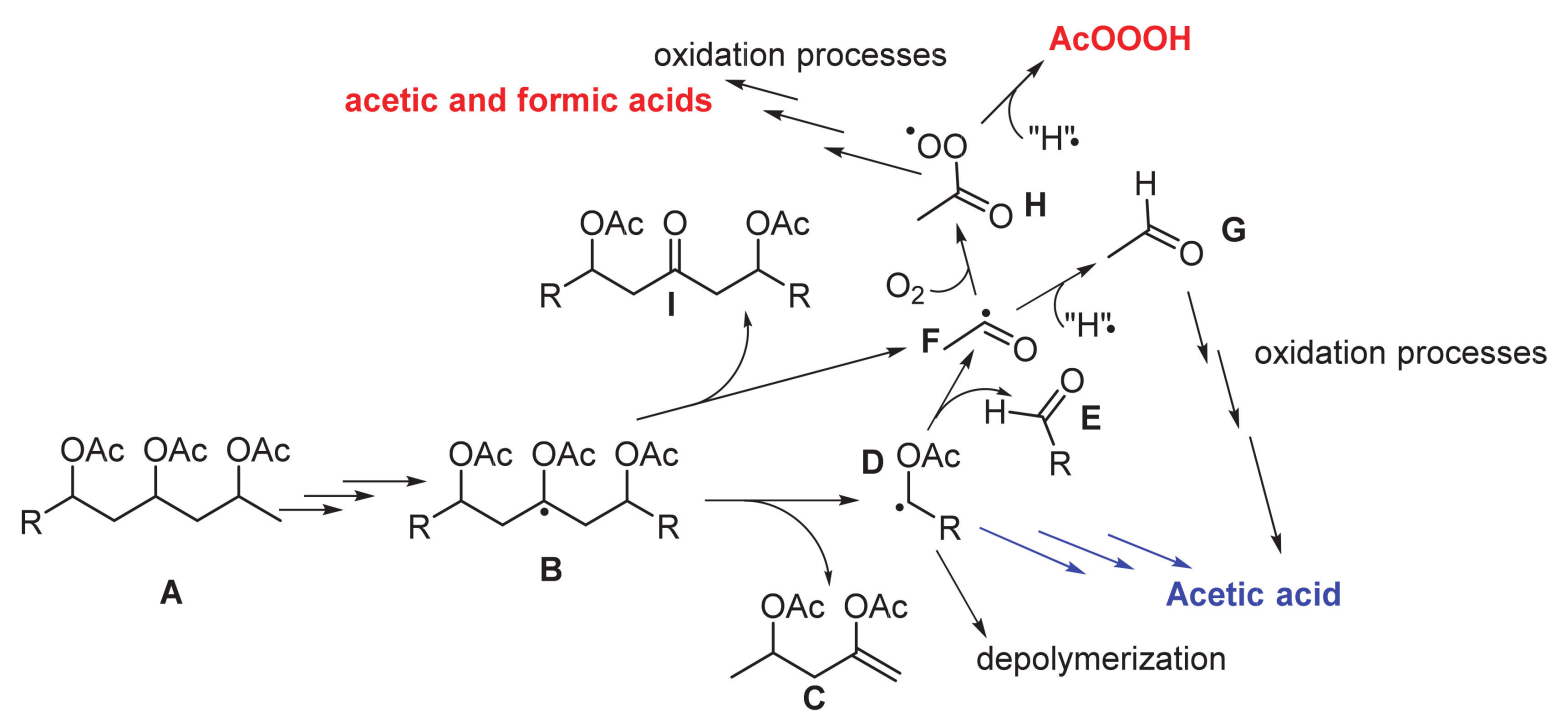
Pathway of peracetic acid generation from the EVA layer. For more details see Figure S4.

The formation of acetic acid observed during the variation of EVA/EVOH/EVA multilayer films is due to several pathways (see Figure S2, Chart S1 for more details) [7]. Nevertheless, one of these pathways implies the generation of acetyl radical **F** (Scheme 3), which either abstracts an H-atom to yield acetaldehyde, further oxidized to acetic acid, or is scavenged by O_2_ to afford acetyl peroxyl radical **H,** which, in turn, abstracts an H-atom to afford peracetic acid for further oxidation of the methionine. Importantly, the reaction of the most labile H-atom in polymer **A** with **F** and **H** radicals affords alkyl radical **B,** which re-initiates a radical chain leading to the production of a large amount of peracetic acid.

Then, all previous reports on the changes of EVA/EVOH/EVA structure nicely support the generation of peracetic acid, which is able to oxidize a solution of methionine. Therefore, bags built with such materials could lead to the modification of biological solutions containing proteins carrying accessible sites of cysteine, methionine, or prone to oxidation moieties. However, although the mechanism of generation of peracetic acid is now unveiled and its consequences well know, the level of oxidation cannot be predicted, as it depends on parameters such as the irradiation dose, the aging of the empty bag, the storage time of the solution in bags, the buffering conditions and the accessibility of prone to oxidation moieties. As factoring processes require the tuning of several parameters to observe the minimal impact of the generation of peracids, a design of the experiment is applied. Hence, 3 parameters were selected: maximization of the oxidative induction time (Y_1_), minimization of methionine sulfoxide formation rate (Y_2_), and minimization of the methionine sulfoxide concentration (Y_3_).

To reach our aim, we applied the desirability function proposed by Derringer and Suich [29] in which the value of the response (Y_1_, Y_2_ or Y_3_) is transformed into an individual desirability function d_i_(Y_i_), representing the degree of satisfaction, scaled between 0 and 100%: 0% pointing at an unacceptable value of Y_i_ and 100% denoting a completely acceptable value.

Two cross-border cases (case 1 is less stringent than case 2) were selected for the requirements (Table 4), affording three curves of individual desirability functions (Figures S3 and S4).

The ideal situation would be: an infinite oxidative induction time (target for Y_1_ = 10 days), no oxidation rate (target for Y_2_ = 0), and no methionine sulfoxide at the end (target for Y_3_ = 0 µM). In the in-use situation, the minimum oxidative induction time accepted is set at two days (case 1) or three days (case 2) to allow the handling of fluids in single-use systems a couple of days without inducing oxidation. Mid- (e.g., >10 days) or long-term storage (e.g., >30 days) would thus require conditions to get an oxidation rate as low as possible, set between 0–3 µM/day for case 1 and between 0–2 µM/day for case 2. Methionine oxidation was used as a probe to monitor the release of oxidizing agents such as peracetic acid and should be minimized. The minimum concentration was zero and up to 3 µM methionine sulfoxide was accepted in case 1, and up to 1 µM in case 2.

The overall desirability was calculated within the experimental domain, by:

$$D={\left(d_{Y1}\times d_{Y2}\times d_{Y3}\right)}^{1/3}$$

**Figure 9:**
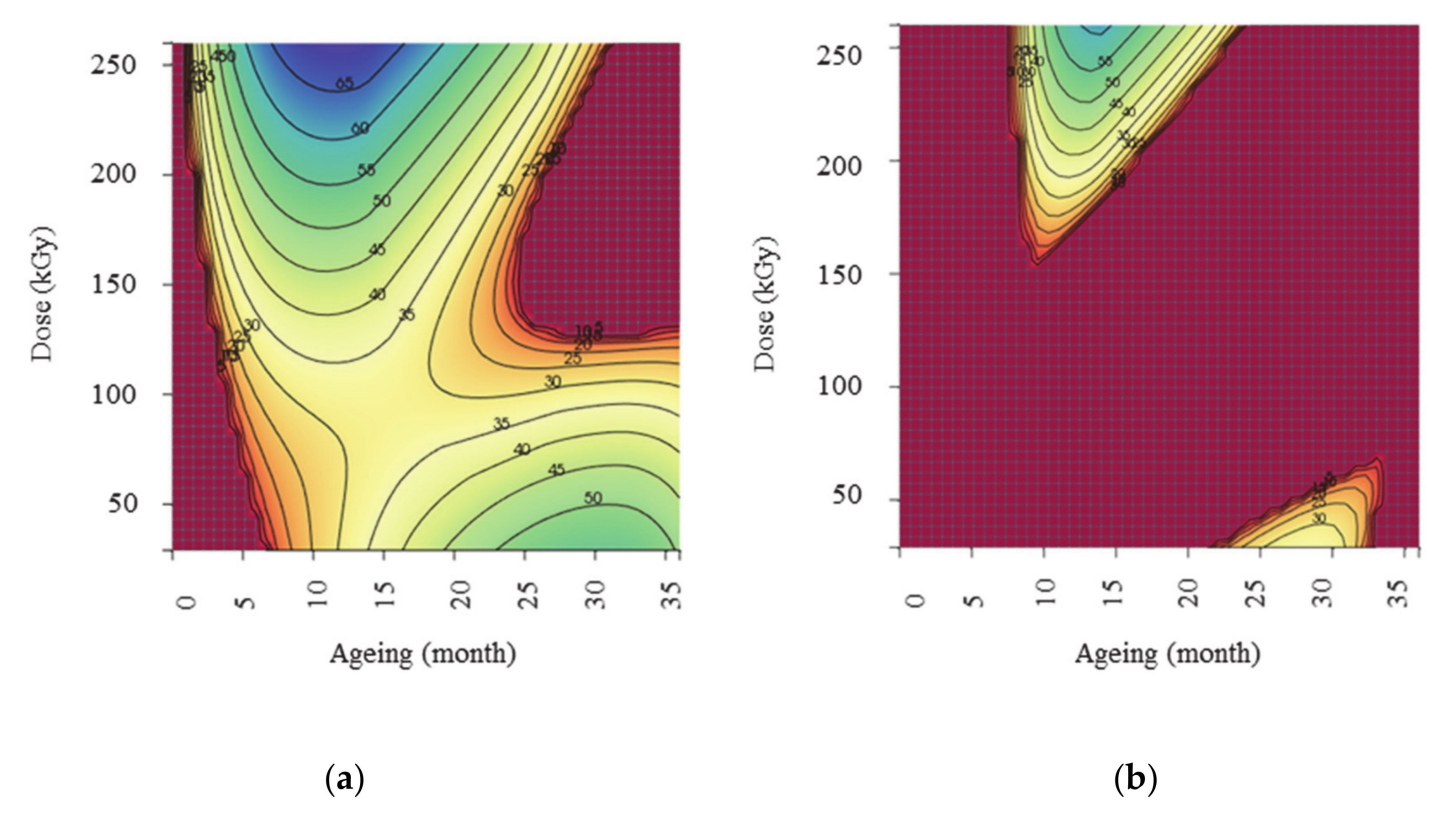
Desirability graphs of aging (months) vs doses (kGy), (**a**) for case 1, (**b**) for case 2.

When an undesirable value was obtained (Y_1_ < value min days or Y_2_ > value max or Y_3_ > value max), the overall desirable value D is 0%, and no compromise was found. In sharp contrast, when each requirement was completely satisfied (Y_1_ ≥ target and Y_2_ ≤ target value and Y_3_ ≤ target value), the overall desirability value was 100%. Finally, when 0% < D < 100%, an acceptable compromise between the different responses was found, as defined in Table 4. The response surface corresponding to desirability function D is displayed in Figure 9a for case 1 and Figure 9b for case 2. The bluish zones represent the highest desirability.

To comply with requirements of case 1—an oxidative induction time longer than 2 days, a methionine sulfoxide formation rate lower than 3 µM/day, and a maximum concentration of methionine sulfoxide detected lower than 3 µM at the same time, and being satisfy a desirability of >50%, two conditions are available:

- Sterilization of bags between 170 kGy and 260 kGy and storage of bags for aging between 5 and 20 months after irradiation
- Sterilization of bags from 30 kGy to 50 kGy and storage of bags for aging between 24 and 36 months after irradiation

To comply with requirements of case 2—an oxidative induction time longer than 3 days, a methionine sulfoxide formation rate lower than 2 µM/day, and a maximum concentration of methionine sulfoxide detected lower than 1 µM at the same time, and being satisfy a desirability >50%—the bags are sterilized from 230 kGy to 260 kGy and stored for aging between 10 and 20 months after irradiation.

The best conditions, in both cases, are: 260 kGy dose and 13-month aging, leading to an oxidative induction time of 5 days, a methionine sulfoxide formation rate of 0.03 µM/day, and a maximum concentration of methionine sulfoxide detected equal to 0.23 µM.

## 5. Conclusions

Methionine oxidation is used to model the gamma radiation-induced changes in single-use bags and thus to monitor peroxide by-products. The mechanism of formation of these reactive species and the methionine oxidation mechanism are described. The methionine sulfoxide formation rate, the oxidative induction time, and the maximum methionine sulfoxide concentration detected were analyzed through a design of experiments. A key aspect of the study is that it highlights that methionine is oxidized through peracetic acid and not necessarily directly by hydro(gen) peroxides. Oxidants (e.g., peracids) generated by the modification of EVA/EVOH/EVA films under gamma irradiation were evidenced and quantified. Moreover, radical pathways for the generation of peracid were proposed. From these investigations, an experimental design was developed as a tool to determine the set of conditions to fine-tune the most appropriate storage conditions. Two sets of conditions were proposed. With the help of reaction kinetics, the optimal conditions for the use of these single-use bags minimizing the impact of radical chemistry were highlighted. The answers to the design of experiments were then considered to obtain the desirability domain to optimize the conditions of use of the single-use bags limiting methionine oxidation and the release of reactive species thereof.

## Figures and Tables

**Table 1:** The domain of interest for the quantitative variables (X1 and X2) of the experimental design.

Variable	Factor	Unit	Quantitative Factors
X_1_	Gamma irradiation dose	kGy	29-59-106-260
X_2_	Aging time	months	0-1-2-3-6-12-24-36

**Table 2:** Details of the different tests to quantify methionine oxidation.

		Sulfoxide Methionine Concentration (µM)								
	Time	Experiment 1	Experiment 2	Experiment 3	Experiment 4	Experiment 5	Experiment 6	Experiment 7	Time	Experiment 8
Conditions		(1) Met ^a^ 50 µM	(1) H_2_O_2_ 50 µM	(1) AA ^b^ 50 µM	(1) H_2_O_2_ 30 µM	(1) H_2_O_2_ 25 µM	(1) Met ^a^ 100 µM	(1) H_2_O_2_ 50 µM		(1) AA ^b^ 256 mM
		(2) H_2_O_2_ 50 µM	(2) AA ^b^ 50 µM	(2) Met ^a^ 50 µM	(2) Met ^a^ 100 µM	(2) AA ^b^ 25 µM	(2) AA ^b^ pH 4,9	(2) Met ^a^ 50 µM		(2) H_2_O_2_ 76 mM
			(3) Met ^a^ 50 µM			(3) Met ^a^ 100 µM	(3) H_2_O_2_ 25 µM			(3) Met ^a^ 50 µM
	0 days	0.4	0.0	0.0	0.6	0.5	0.4	0.6	20 min	0.0
	3 days	7.1	4.3	0.0	7.2	6.5	3.5	7.5	60 min	3.7
	14 days	20.0	12.0	0.4	20.7	18.6	10.2	21.5	80 min	4.3
	21 days	18.3	16.4	0.4	23.0	20.6	11.6	26.4	100 min	4.2
									120 min	5.0
									720 min	6.6

^a^ Met, methionine; ^b^ AA, acetic acid.

**Table 3:** Coefficients obtained from experimental results.

Coefficient	Y_1_ Oxidative Induction Time	Y_2_ Methionine Sulfoxide Formation Rate (µM/day)	Y_3_ Maximum Concentration of Methionine Sulfoxide Detected
b_0_	2.30	−0.10	−0.32
b_1_	0.58	−0.02	−0.40
b_2_	−1.02	−0.27	−1.23
b_11_	1.06	0.07	0.47
b_22_	0.53	0.59	3.07
b_12_	−2.01	0.19	0.70

**Table 4:** Requirements values for each response, in case 1 and 2.

	Case 1	Case 2
**Y_1_ (days)** Oxidative induction time	Min: 2 Target: 10	Min: 3 Target: 10
**Y_2_ (µM/day)** Methionine sulfoxide formation rate	Target: 0 Max: 3	Target: 0 Max: 2
**Y_3_ (µM)** Methionine sulfoxide concentration	Target: 0 Max: 3	Target: 0 Max: 1

## Supplementary Materials

The following are available online at https://www.mdpi.com/2073-4360/12/12/3024/s1, Table S1. automated online derivatization, Table S2. Experimental design and results. Figure S1. Desirability functions for oxidative induction time (dY1) (days), methionine sulfoxide formation rate (dY2) and maximum concentration of methionine sulfoxide detected (dY3) (µM) for D1, Figure S2. Desirability functions for oxidative induction time (dY1) (days), methionine sulfoxide formation rate (dY2) and maximum concentration of methionine sulfoxide detected (dY3) (µM) for D2, Figure S3. Methionine sulfoxide concentration in function of time of the seven different tests to observe methionine oxidation, Figure S4. Radicals processes generated by γ-irradiation accounting for chemical and physical modification observed in the materials. Chart S1. list of intermediates present in the Figure S4.
