# Supplementary material for: Monitoring of Peroxide in Gamma Irradiated EVA Multilayer Film Using Methionine Probe
Source: Polymers (Basel). 2020 Dec 17;12(12):3024. doi: 10.3390/polym12123024 (PMC7766525; doi:10.3390/polym12123024)
Supplement: Supplementary file 1 [file polymers-12-03024-s001.pdf]

Supporting information

Monitoring of peroxide in gamma irradiated EVA multilayer film using methionine probe

Nina Girard-Perier[1,2,3], Magalie Claeys-Bruno[2], Sylvain R.A. Marque[3], Nathalie Dupuy[2], Fanny Gaston[1], Samuel Dorey[1]

1 Sartorius Stedim FMT S.A.S, Z.I. Les Paluds, Avenue de Jouques CS91051, 13781 Aubagne Cedex, France
2 Aix Marseille Univ, Avignon Université, CNRS, IRD, IMBE, Marseille, France
3 Aix Marseille Univ, CNRS, ICR, case 551, 13397 Marseille, France

Corresponding authors:
samuel.dorey@sartorius.com
m.claeys-bruno@univ-amu.fr
nathalie.dupuy@univ-amu.fr
sylvain.marque@univ-amu.fr

Table S1. automated online derivatization

1. Draw 2.5 µL from borate vial
2. Draw 1.0 µL from sample vial
3. Mix 3.5 µL in wash port five times
4. Wait 0.2 minutes
5. Draw 0.5 µL from OPA vial
6. Mix 4.0 µL in wash port 10 times default speed
7. Mix 4.4 µL in wash port 10 times default speed
8. Draw 32 µL from injection diluent vial (100 mL of mobile phase A and 0.4 mL concentrated H_3PO_4)
9. Mix 20 µL in wash port eight times
10. Inject
11. Wait 0.1 minutes
12. Valve bypass

Table S2. Experimental design and results

Dose (kGy)	Ageing (Months)	Lot	Oxidative induction time (days)	Methionine sulfoxide formation rate	Maximum concentration of methionine sulfoxide detected (µM)
29	0	1	1	2.15	8.4
29	0	2	1	2.15	7.26
29	1	1	3	0.35	3.85
29	1	2	3	0.38	3.78
29	2	1	3	0.47	4.46
29	2	2	3	0.47	4.11
29	3	1	3	0.99	3.86
29	3	2	3	0.26	2.99

29	6	1	3	0.14	1.51
29	6	2	3	0.14	1.55
29	12	1	3	0.15	1.35
29	12	2	3	0.15	1.36
29	24	1	3	0.11	0.71
29	24	2	3	0.13	0.76
29	36	1	3	0.07	1.33
29	36	2	3	0.08	1.52
59	0	1	1	2.18	8.3
59	0	2	1	2.37	7.41
59	1	1	1	0.76	5.31
59	1	2	3	0.53	6.34
59	2	1	3	0.44	4.6
59	2	2	1	1.04	4.69
59	3	1	3	0.33	2.93
59	3	2	3	0.35	2.96
59	6	1	3	0.18	2.89
59	6	2	3	0.17	1.66
59	12	1	3	0.14	1.52
59	12	2	3	0.12	1.27
59	24	1	3	0.11	0.46
59	24	2	3	0.11	0.64
59	36	1	3	0.06	1.5
59	36	2	3	0.08	1.48
106	0	1	1	1.99	5.29
106	0	2	1	1.3	4.61
106	1	1	3	0.31	2.99
106	1	2	3	0.26	2.67
106	2	1	3	0.42	3.36
106	2	2	3	0.38	3.17
106	3	1	3	0.23	1.84
106	3	2	3	0.24	1.97
106	6	1	3	0.13	1.18
106	6	2	3	0.13	1.09
106	12	1	3	0.19	1.62
106	12	2	3	0.19	1.51
106	24	1	3	0.09	0.21
106	24	2	3	0.1	0.59
106	36	1	3	NA	NA
106	36	2	3	0.05	1.28
260	0	1	10	0.32	4.43
260	0	2	10	0.33	4.13
260	1	1	10	0.5	3.9
260	1	2	10	0.48	3.84
260	2	1	10	0.34	1.81
260	2	2	10	0.37	2.02
260	3	1	3	0.18	0.78
260	3	2	3	1.12	0.92

260	6	1	3	0.12	NA
260	6	2	3	0.3	1.44
260	12	1	3	0.48	NA
260	12	2	3	0.5	1.03
260	24	1	3	0.08	0.78
260	24	2	3	0.03	0.88
260	36	1	3	0.05	0.83
260	36	2	3	0.05	NA

All the experiments have been performed in duplicate allowing the calculation on the experimental variance for each response, equal to 0.1, 0.040 and 0.12 respectively with 31, 29 and 28 degrees of freedom.

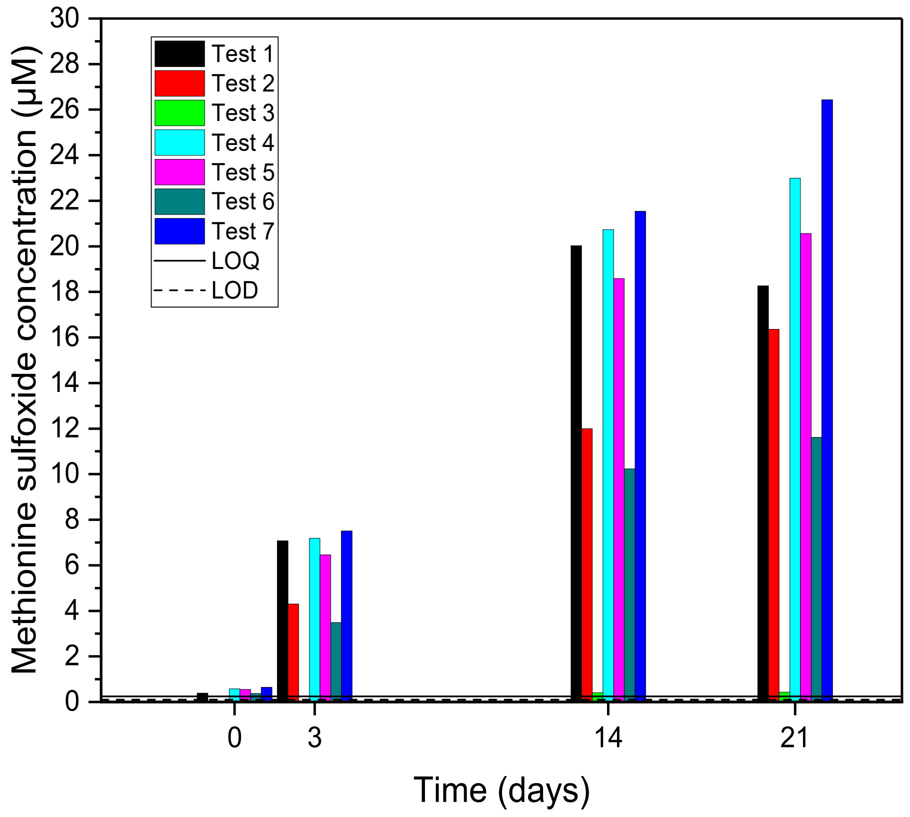

Figure S1. Methionine sulfoxide concentration in function of time of the seven different tests to observe methionine oxidation. It has to be mentioned that these model experiments cannot reproduce the concentrations of H_2O_2 and acetic acid released in solution by the material because the rates of migration of molecules in materials are not known, and the concentration generated cannot be controlled and estimated.

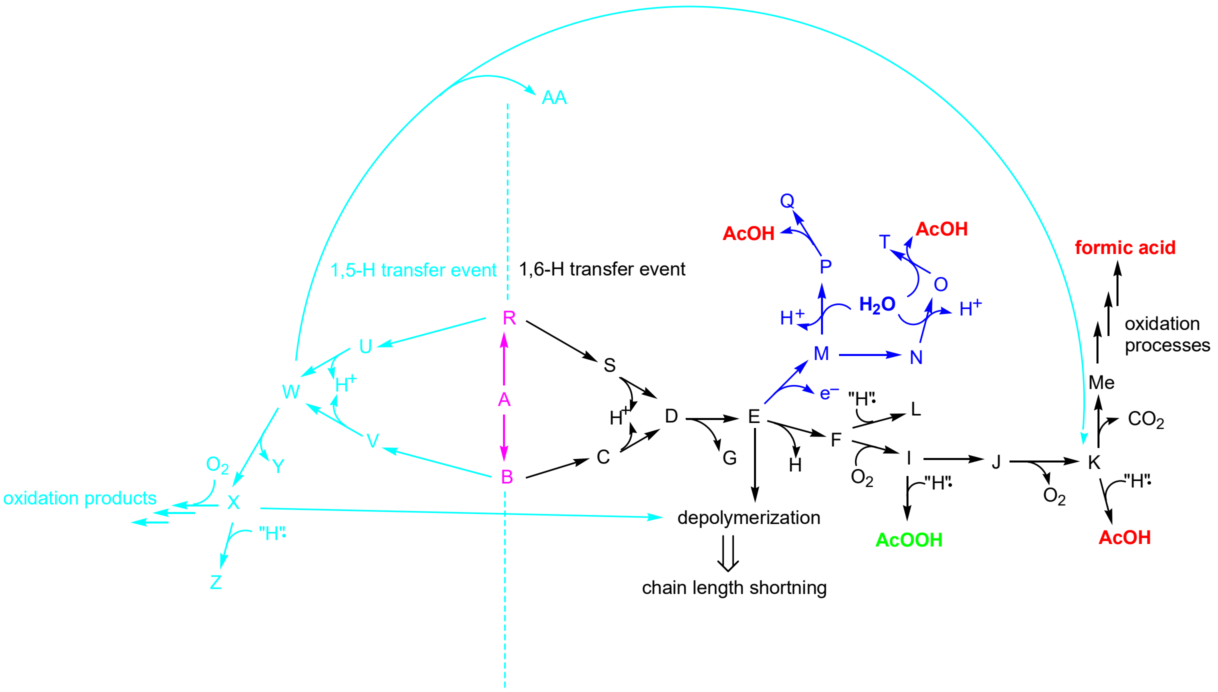

Figure S2. Radicals processes generated by γ-irradiation accounting for chemical and physical modification observed in the materials

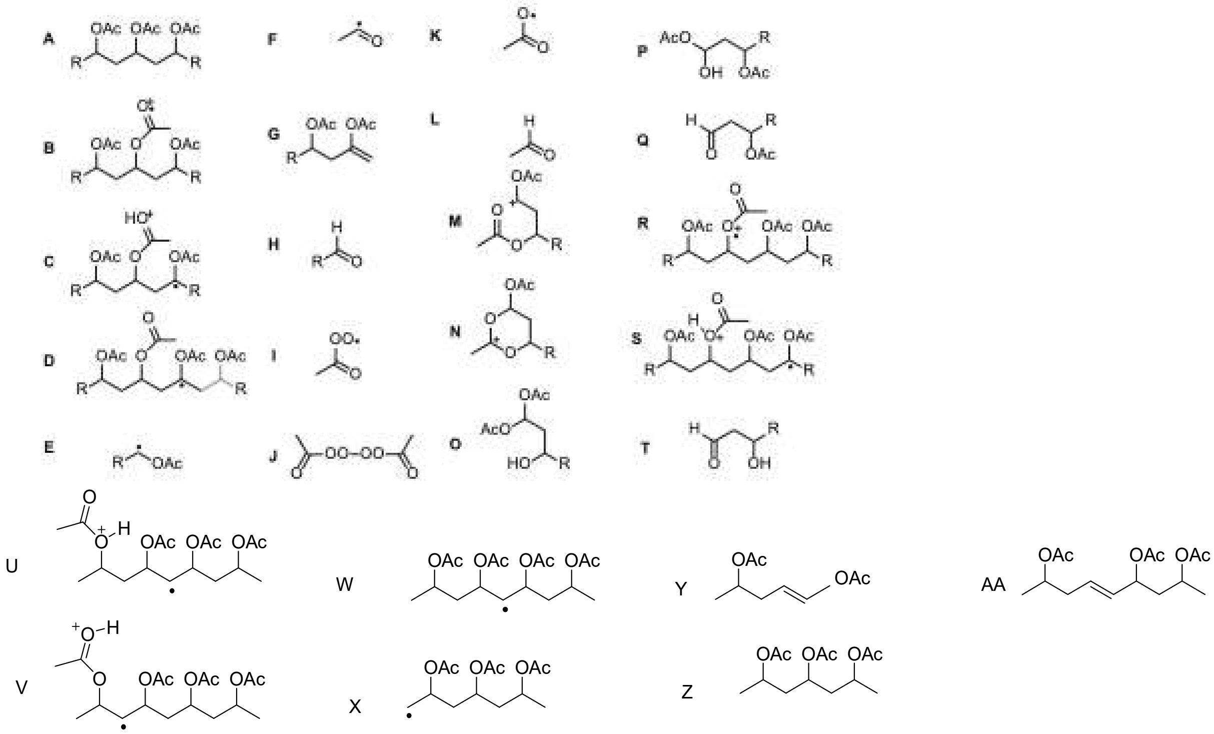

Chart S1. list of intermediates present in the Figure S2

$\underline{d_{Y1}}$

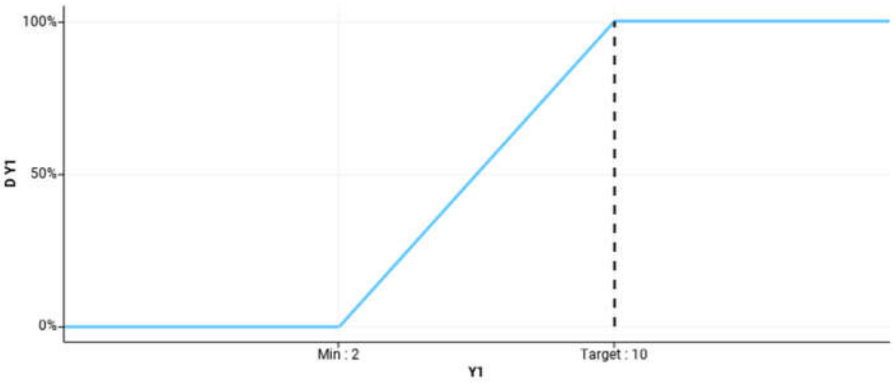

$\underline{d_{Y2}}$

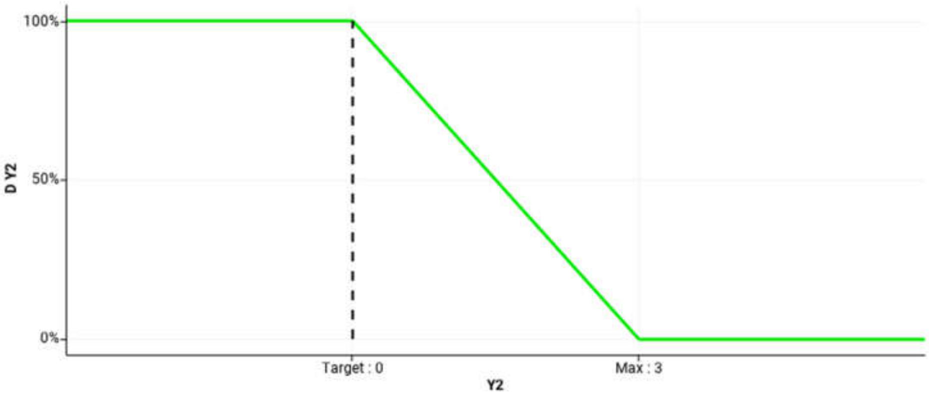

$\underline{d_{Y3}}$

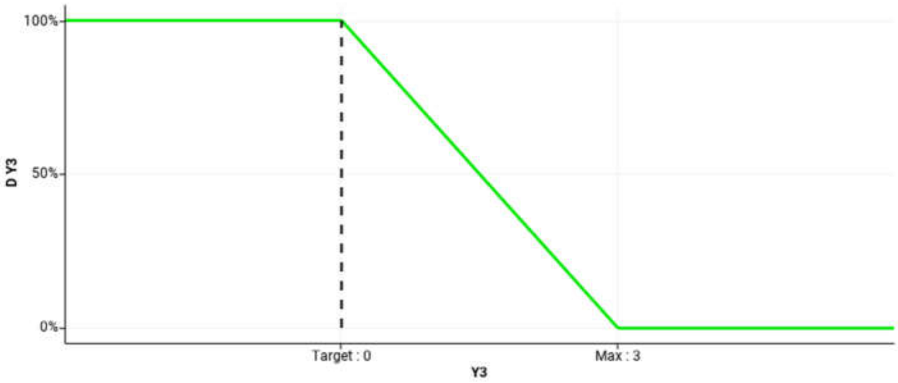

Figure S3. Desirability functions for oxidative induction time (d_{Y1}) (days), methionine sulfoxide formation rate (d_{Y2}) and maximum concentration of methionine sulfoxide detected (d_{Y3}) (μM) for D1

d_{Y1}

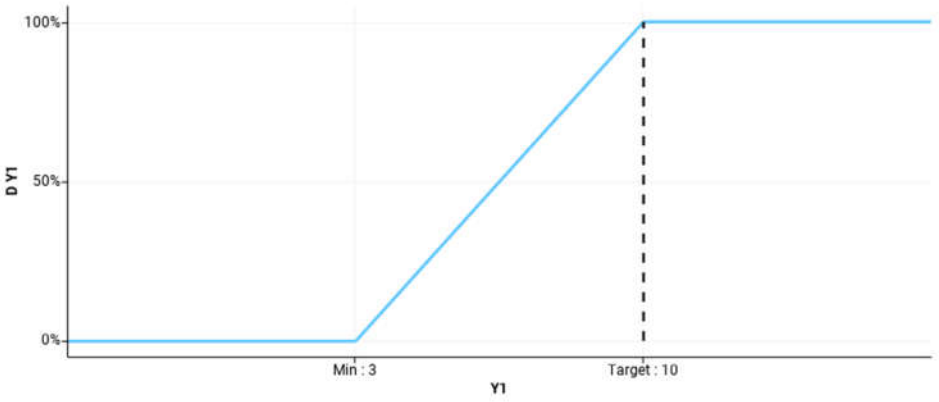

d_{Y2}

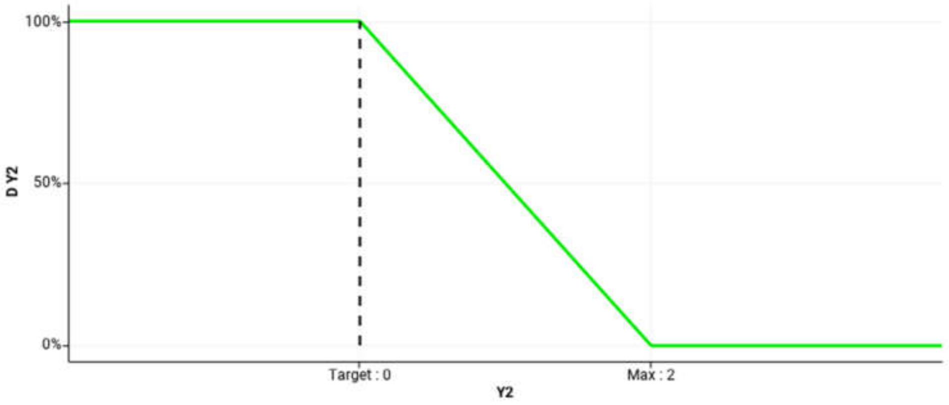

d_{Y3}

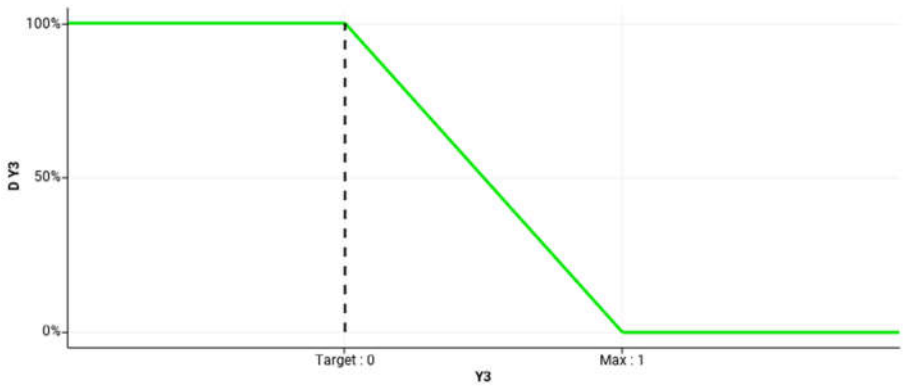

Figure S4. Desirability functions for oxidative induction time (d_{Y1}) (days), methionine sulfoxide formation rate (d_{Y2}) and maximum concentration of methionine sulfoxide detected (d_{Y3}) (μM) for D2
